# Technological, institutional, and geographical peripheries: regional development and risk of poverty in the European regions

**DOI:** 10.1007/s00168-022-01127-9

**Published:** 2022-04-02

**Authors:** Javier Barbero, Ernesto Rodríguez-Crespo

**Affiliations:** 1 European Commission, Joint Research Centre (JRC), Seville, Ed. EXPO, C/Inca Garcilaso 3, 41092 Seville, Spain; 2 Department of Economic Structure and Development Economics, Universidad Autónoma de Madrid, C/Francisco Tomás Y Valiente, 5, 28049 Madrid, Spain

**Keywords:** R11, O15, O43, D63

## Abstract

In this paper, we analyze the interrelation between technological, institutional, and geographical peripheries. By distinguishing between the quality and quantity of access to information and communication technologies, together with institutional and geographical factors, and using a sample of 229 European regions during the period 2007–2018, we find that the diffusion and quality of information and communication technologies foster economic development and decrease the risk of social exclusion. A similar effect is found for institutional and geographical factors, suggesting that the interplay of these three determinants may be crucial to set up place-based policies.

**Supplementary Information:**

The online version contains supplementary material available at 10.1007/s00168-022-01127-9.

## Introduction

The interplay between technology, geography, and institutions has been proved to affect not only the ongoing process of globalization, but also past events of profound economic change (Sachs 2020), and these factors impact economic development. This fact may be particularly crucial at the regional level, especially for the case of Europe, where several institutional efforts have been taken to reduce disparities between European countries. However, we still find salient and pronounced differences between core and periphery regions between and within European Union Member States. These differences have been widely studied and explained by factors related to geography, recognizing the importance of accessibility, factor endowments, and being closer to the economic core to explain the growth and economic development of the regions (e.g., Krugman 1991; Fujita et al. 1999). Digital technologies, which can be applied to low-productivity sectors by small and medium enterprises, and improvements in social inclusion for European citizens are among the drivers that can reduce territorial inequalities (European Commission 2018, 2020). Finally, it has also been recognized that there exist sharp differences in the quality of government at the regional level (Charron et al. 2014), and that government quality play is an important role in regional prospects (Ketterer and Rodríguez-Pose 2018).

Despite identifying these determinants, the dynamics of regional inequalities in the European Union tend to persist over time. The most recent report on the effect of national policies and cohesion in the European Union has found between and within-country inequalities that persist in the European Union and different drivers to reduce these inequalities (European Commission 2017). More recently, inequalities have upsurged due to the COVID-19 pandemic, raising spatial differences in Europe (e.g., Florida and Mellander 2020; Rodríguez-Pose and Burlina 2021). After the diffusion of the pandemic, access to technology has become a must, as many firms in European countries opted to implement working at home. At the same time, schools and universities continued their teaching activities using online platforms. Dingel and Neiman (2020), using pre-pandemic data, find that jobs that can be performed at home account for a large share of value added—wages, and that lower-income countries have a lower share of jobs that can be done remotely. Therefore, the economy of regions with less deployment of ICT infrastructure and illiteracy in digital skills might suffer more due to the stringency measures taken by the government to stop the spread of the virus.

The traditional and dominant strategies to address territorial inequalities have considered physical and human capital, together with technology, but this has not been successful in reducing territorial inequalities (Rodríguez-Pose 2020). The simultaneous consideration of technology, institutions, and geography at the subnational level can shed light on how to understand regional development dynamics. In parallel, inequality is particularly relevant, as it may constitute a threat to political stability and social cohesion at the regional level (Iammarino et al. 2019). Concerning previous studies, we acknowledge that the impact of technology (Martínez and Rodríguez 2009), and geography and institutions (Ketterer and Rodríguez-Pose 2018; Rodríguez-Pose and Ketterer 2020) has been studied separately and restricting to economic growth. The literature on regional inequalities in the European Union usually neglects the impact of information and communication technologies (Perugini and Martino 2008; Royuela et al. 2019), even though the impact of ICT on inequality depends on the type of ICT and the measure of inequality at country level (Richmond and Triplett 2018). This statement is even more important at the subnational level, given the pronounced differences between developed and lagging regions (McCann and Ortega-Argilés, 2015). These findings suggest that evidence is far from conclusive and demand to consider them to shed light on the dynamics of regional development.

This study follows a holistic approach integrating previous studies by assuming that technology, institutions, and geography affect regional economic development and regional inequalities. We use panel data econometric techniques to analyze the determinants of economic growth and study which determinants of regional economic prosperity are also related to the risk of poverty or social exclusion, which constitutes a relevant measure of inequality.

Using a sample of 229 European Union regions during the period 2007–2018, we find that both the diffusion and quality of information and communication technologies, the quality of institutions, and the geography foster economic development and decrease the risk of poverty and social exclusion. Our results reinforce the importance of place-based policies to reduce the rising inequality in peripheral regions since their lower endowments of information and communication technologies, institutional quality, and accessibility affect aggregate performance and may be considered among the sources of the higher level of inequality and social exclusion.

This paper is organized as follows. Section 2 explains the theoretical and conceptual framework, while Sect. 3 develops the literature review. Section 4 describes the empirical analysis, and Sect. 5 shows the main results. Finally, Sect. 6 concludes by highlighting policy recommendations and avenues for future research.

## Explaining economic growth and development at the regional level: why regions matter

### The impact of digital technologies on growth and inequality

Digital technologies have completely reshaped different fields of analysis, such as the social, economic, and geographical contexts. Among the multiple effects of digital technologies, economic and productivity growth is always cited as the most prominent, both at the country (Cardona et al. 2013) and regional level (Karlsson et al. 2010).

Digital technologies are unevenly distributed across space, a fact that has created between and within-country inequalities known as the digital divide (James 2003). In this process, developed countries show higher rates of ICT diffusion compared to developing countries. In addition, the digital divide increases in parallel to geographical disaggregation. This digital divide is as an additional source of inequality between regions and demands urgent policy actions.

**Fig. 1:**
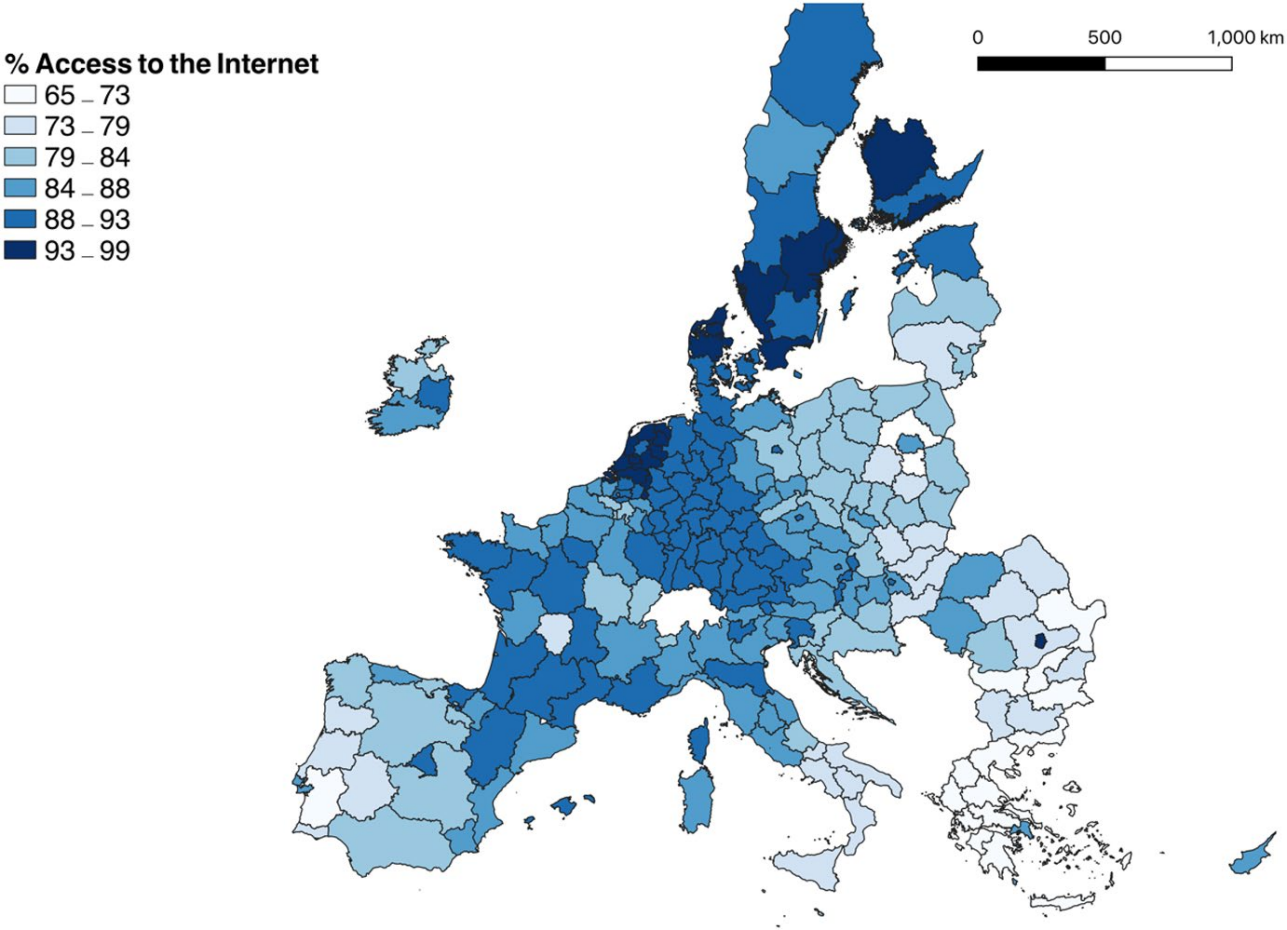
Percentage of households with access to the Internet at home in 2018. *Source* Eurostat. EuroGeographics for the administrative boundaries. The continental EU regions of Pohjois- ja Itä-Suomi (Finland) and Mazowiecki regionalny (Poland) are excluded

Figure 1 displays the percentage of households with access to the Internet at home in 2018, showing the existence of asymmetric diffusion levels of Internet access across the European Union. The lower levels of access to the Internet in Eastern Europe, Greece, southern Italy, and Portugal, compared to central and northern Europe, reveal the existence of technological peripheries.

Measuring ICT using the percentage of subscriptions is the traditional measure, but this variable evolves with population and does not capture the quality of ICT connectivity because it may be subject to saturation (Hilbert 2016). To this end, it is necessary to complement Internet users with other ICT variables, such as access to broadband, which is more intrinsically related to the quality of subscriptions. Broadband offers greater opportunities to work remotely, and to consume digital services from home—such as video streaming services. The percentage of broadband subscriptions in the European Union in 2018 showed that certain northern regions, like Groningen, reached almost 100%, while southern regions seemed to exhibit a lower diffusion rate of Internet compared with northern regions. See Fig. A1 in the Appendix for a map of the percentage of households with broadband access.

### Unveiling the importance of institutional quality

Country-level data have helped address the growth differences between countries and the role of potential growth determinants, especially in the fields of economics and geography. However, country-level data cannot capture within-country variations of regional growth levels and may not be useful in formulating policies of regional development.

From the middle of the 1990s, regional statistics have been collected by different international institutions and national offices of statistics. The European Union is one of the geographical areas where more efforts have been made to elaborate regional statistics for member states for different levels of territorial disaggregation. Although regional statistics tend to be scarcer than country-level statistics, they provide a better understanding of interregional and intraregional growth disparities and contribute to formulating more accurate policies of regional development. In the case of the European Union, the impact assessment of the Cohesion Policy, whose aim is to reduce regional inequalities, has become one of the main priorities, and regional statistics can contribute to evaluating whether regional inequalities are being reduced.

Growth is intrinsically related to the dynamics of regional development. According to neoclassical economics, inequality is transitory since those regions with lower growth levels will surpass, in the long run, the regions with higher growth levels in order to reach convergence—growth rates tend to equalize across regions (Barro and Sala-i-Martin 2003).

Among the elements that may contribute to balance growth rates in the long run and hence alter economic development, the issue of institutional quality is particularly relevant. According to the neoclassical growth theory, an increasing part of the residuals in the economic growth models suggest that there may be additional determinants beyond capital and labor, such as institutional quality (Rodríguez-Pose 2020). The quality of institutions depends on the impact on economic performance, as there may be detrimental institutions to accumulate human capital and technology, resulting in lower economic development (Acemoglu and Robinson 2012). However, the study of the impact of institutions on economic performance is certainly controversial due to the following reasons. First, a universal definition of institutions is absent from the analysis and the discussion (Nunn and Trefler 2014). Second, institutions are found to depend on time and context (Rodríguez-Pose 2013), requiring finding a suitable measure on institutional quality. Third, measures of the quality of institutions at the subnational level are scarce compared to the country level, but recent attempts have estimated regional measures of quality of government, such as Charron et al. (2014) for European regions.

### The European union: the persisting difference between developed and lagging regions

**Fig. 2:**
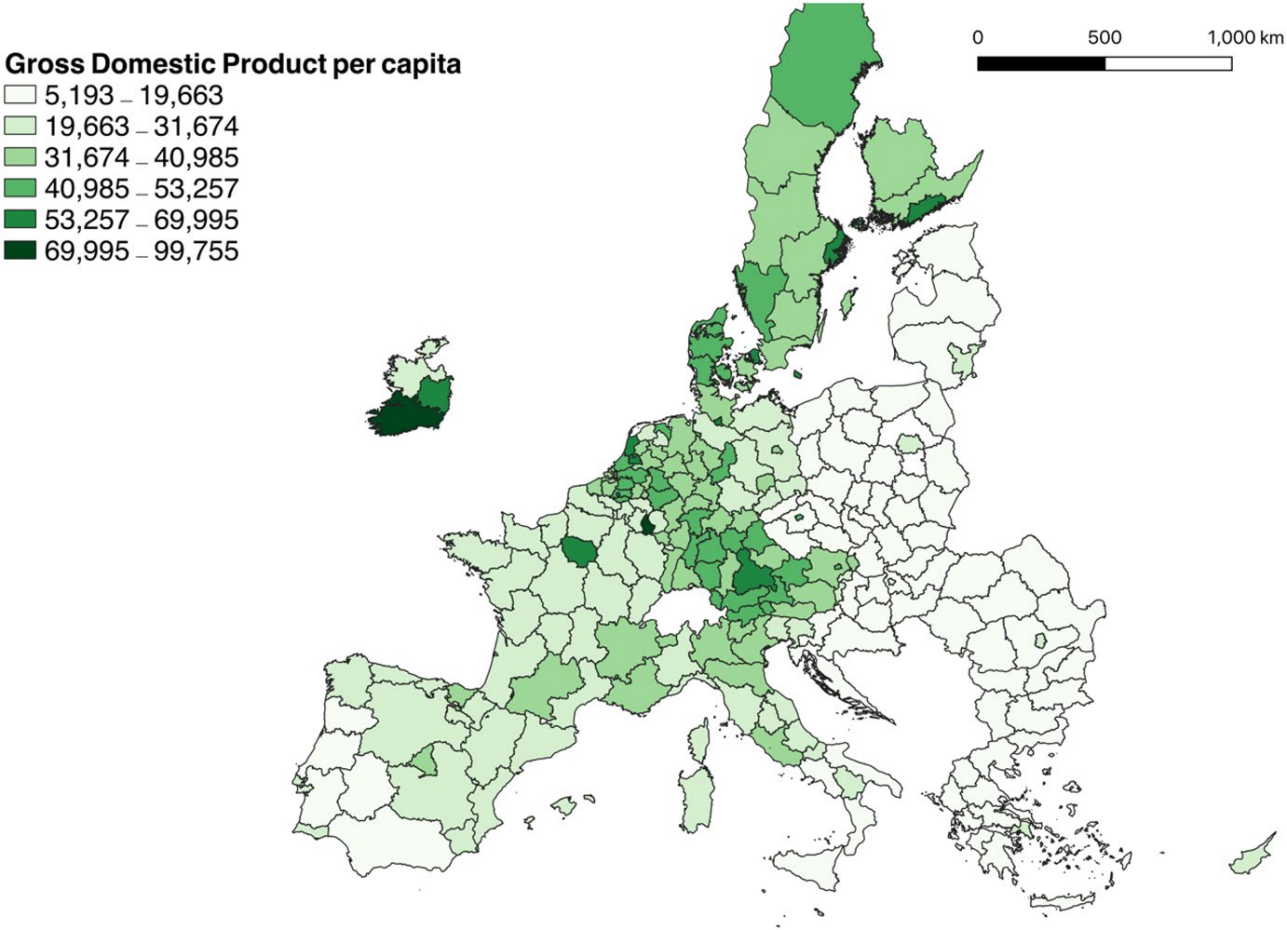
Gross domestic product (GDP) per capita in the European Union in 2018. *Source*: Eurostat. EuroGeographics for the administrative boundaries. The continental EU regions of Pohjois- ja Itä-Suomi (Finland) and Mazowiecki regionalny (Poland) are excluded

Despite the Cohesion Policy aimed to reduce regional disparities in the European Union, the difference between more developed and less developed regions is becoming widespread (European Commission 2018, 2020). Figure 2 shows the gross domestic product (GDP) per capita for all EU regions in 2018, revealing the existence of within and between country differences. Regions in Eastern Europe, Greece, southern Italy, and southern Spain and Portugal have the lowest levels of economic prosperity.

**Fig. 3:**
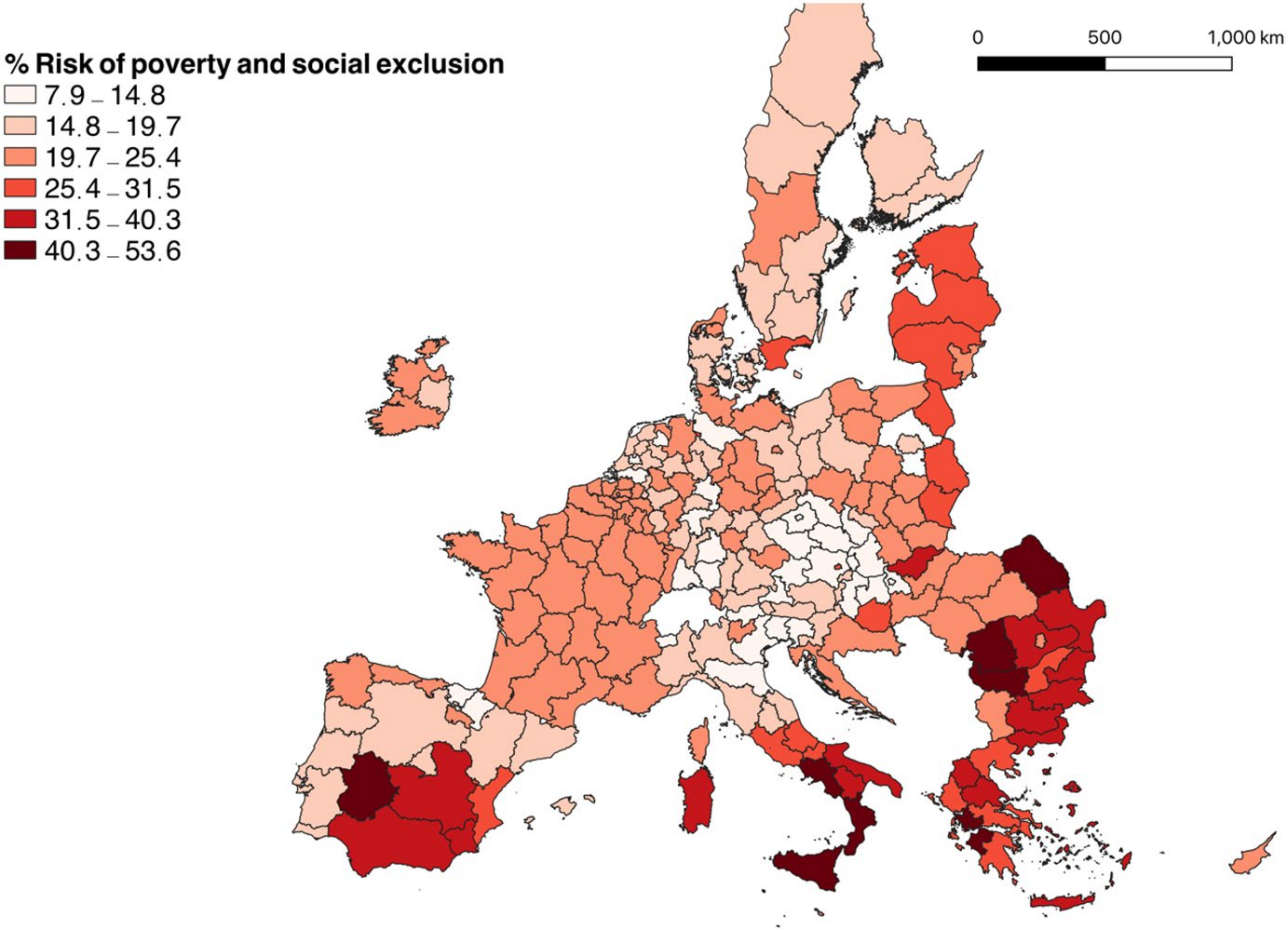
People at risk of poverty or social exclusion in the European Union, 2018. *Source* Eurostat. EuroGeographics for the administrative boundaries. The continental EU regions of Pohjois- ja Itä-Suomi (Finland) and Mazowiecki regionalny (Poland) are excluded

Figure 3 displays the percentage of people at risk of poverty or social exclusion in the European Union in 2018. Northern regions show the lowest rates of social exclusion. In contrast, populations in Eastern Europe, Greece, southern Italy, and southern Spain are at higher risk of poverty and social exclusion. Looking simultaneously at Figs. 2 and 3, we can observe that regions with lower GDP per capita also have a higher percentage of population at risk of poverty and social exclusion.^1^ This reveals that those regions are not only less developed, but also individuals in those places are at higher risk of being excluded from society.

The European Union has acknowledged the existence of regional disparities and the importance of the Cohesion Policy as the main driver to address these disparities. According to the Cohesion Policy, regions are classified under three Objectives according to their priority for receiving Cohesion Funds. The least developed regions are considered as part of Objective 1 with higher priority, while middle and high-income regions are part of Objectives 2 and 3, respectively. Several factors can contribute to explaining the growth differences between European regions. Differences in infrastructure and technology are cited among the most important to explain differences in European regional economic development, since lagging regions exhibit lower levels of infrastructure and penetration of new technologies (McCann and Ortega-Argilés, 2015) giving rise to a regional divide in terms of access to ICT (Vicente and López 2011; Crespo-Cuaresma and Lutz 2021).

Designing effective policy actions is key to reducing regional disparities in the European Union, especially in the long run. Neutral policies, without differentiating according to the characteristics of the territories, have constituted the most common approach, but recent approaches suggest that place-based policies may be more effective in reducing regional disparities (Iammarino et al. 2019; Rodríguez-Pose 2020).

The implementation of place-based policies, however, needs to be carefully planned, since the amount of investment needs to be complemented with an appropriate environment to obtain the returns on the investment. As demonstrated by Rodríguez-Pose and Wilkie (2019) for the case of human capital, investments without the creation of a local environment and jobs may result in brain drain and the problem will remain unsolved. The role of institutions is crucial since places with higher levels of regional growth and development tend to have inclusive institutions.^2^

New approaches suggest that regional disparities in the European Union may be reduced by a twofold approach: reinforcing the strongest regions but, at the same time, fostering lagging regions, resulting in a novel “place-sensitive distributed development policy” (Iammarino et al. 2019). Hence, it is essential to differentiate between rich and lagging regions—regions with low income or low growth—when studying patterns of regional development in the European Union.

Table 1 shows the average values for some key variables for both developed and lagging regions in 2016.^3^ A comparison of the average between the two groups of regions reveals that lagging regions are, on average, less developed, with higher risk of poverty, with lower access to the Internet and lower quality connections, are less educated, with weaker institutions, and less accessible. That is, lagging regions are, at the same time, economic peripheries, social peripheries, technological peripheries, and geographical peripheries.

**Table 1:** Average key economic, social, technological, and geographical variables for developed and lagging regions in 2016

	Developed	Lagging
GDP per capita (€)	29,745.414	14,271.436
Risk of poverty (%)	21.957	35.832
Households internet (%)	83.954	71.035
Household broadband (%)	80.826	70.085
Share employment ICT (%)	2.719	1.268
Institutions	0.322	− 0.877
Heating degree days	2,842.624	1,725.094
Accessibility	0.401	0.254
Ruggedness	2.030	2.770
Investment (million €)	12,362.642	4,317.371
Population (number)	1,953,119.145	1,705,588.083
Human capital (%)	30.034	22.147

## Review of empirical work

Previous empirical work has usually considered the impact of geographical, institutional, and technological factors on economic growth per se. Although the evidence is quite prolific at the country level confirming the positive impact of these determinants on economic growth, as for the case of institutional quality (e.g., Glaeser et al. 2004; Acemoglu and Robinson 2008), information technology (e.g., Jorgenson and Stiroh 1999; Niebel 2018) and also geography (e.g., Nunn and Puga 2012; Sachs 2018) they tend to analyze this set of determinants separately.^4^ As we alleged previously, evidence at the regional level is relatively scarce compared to country level, mainly due to the lack of available statistics at the subnational level. Still, different studies have aimed to shed light on the impact of the aforementioned factors, always focusing on regional growth dynamics. However, either such determinants are considered separately, or they focus on single countries.

To this end, the first set of studies considered technology and R&D activities as the main factors that may lead lagging regions to increase growth. Despite the consideration of technology as a growth driver, studies on ICT use at the regional level have not considered the existence of heterogeneous agents (i.e., households, firms, and governments) that may result in different ICT trajectories and asymmetric regional growth (Billon et al. 2017). Martínez and Rodríguez (2009) study the impact of ICT-related equipment on regional economic growth for the case of Andalusia, a Spanish Southern region, during the period between 1995 and 2006 and find that ICT has contributed to increasing both GDP and labor productivity. Stephens et al. (2013) analyze the impact of innovation and entrepreneurship on economic growth for 135 lagging US states during the period 1990–2007. Although they find a positive impact of entrepreneurship and creativity on economic growth, other factors related to technology and human capital do not seem to foster growth in lagging regions. Salemink et al. (2017) develop a systematic literature review on the effect of ICT availability in rural areas and find that ICT is a growth facilitator and that rural areas require higher levels of ICT connectivity. However, they are the areas registering lower rates of ICT diffusion and adoption. For the specific case of the growth dynamics of the European Union, Gagliardi and Percoco (2017) carry out a policy impact to assess whether the Cohesion Policy in the European Union has mitigated regional disparities. Using a sample of 257 European regions for the period 2000–2006, they conclude that the Cohesion Policy has fostered growth in lagging regions. However, this growth is conditioned to geography, since growth rates are higher for those lagging regions located closer to urban agglomerations.

The second set of studies has assumed that increasing the quality of institutions, measured by the quality of government, might be the key factor explaining differences between advanced and lagging regions. Rodríguez-Pose and Ketterer (2020) study the effect of institutional quality on regional economic growth for a sample of 249 European regions during the period 1999–2013. By distinguishing between developed and lagging regions, they find that lagging regions can benefit more from increasing the quality of their institutions. Rodríguez-Pose and Ganau (2021) find that institutional quality matters when explaining differences in labor productivity for European regions during the period 2003–2015. However, information technology and digitalization are absent from the discussion in their analysis at the European Union subnational level.

Other studies have analyzed the factors mentioned above focusing on developed regions or a set of developed and lagging regions. Van Gaasbeck (2008) used data for a developed US region, California, for the period 2001–2006 and finds that broadband access has contributed to increasing overall economic growth and employment. Ketterer and Rodríguez-Pose (2018) analyze whether the economic growth in a sample of 172 European regions during the period 1995–2009 is driven by geography or institutions. They find institutions more important than geography, as fighting against corruption may result in more growth, although the importance of geography cannot be neglected.

As similar growth rates may affect poverty differently (Department for International Development 2008), it is necessary to complement our analysis beyond regional development. Consequently, the impact of these growth drivers on inequality also needs to be considered to complement the explanation of regional growth dynamics. Few studies have addressed the explanatory factors of inequality at the regional level, where lack of data is also an important problem to overcome. Studies focus on European (Perugini and Martino 2008; Castells-Quintana et al. 2015) or OECD regions (Royuela et al. 2019) and find that economic, social, and institutional determinants matter to explain regional inequality. However, this strand of literature is isolated from growth and development determinants, so they cannot be compared simultaneously. Although ICT impacts on income inequality are well documented at the country level, empirical evidence suggests that they depend on the type of ICT (Richmond and Tripplet 2018), so that the evidence is far from conclusive. This debate may also be extended to the regional level, despite the availability of existing data to measure ICT and income inequality.

Albalate et al. (2021) build a geographical endowment indicator by regressing the log of population density on a set of geographical variables. They find that greater deviation of population distribution from its geographical endowment leads to worse regional growth convergence and higher economic inequality. However, they leave ICT and institutional quality aside from the analysis.

Although the existing previous literature has analyzed the drivers of economic growth with high accuracy and has paid attention to the impacts of human capital, institutional, technological, and geographical determinants of regional growth, we find certain main shortcomings that deserve further empirical attention. First, it has been recognized that the interplay between technology, institutions, and geography has shaped globalization (Sachs 2020) and has therefore affected economic growth. However, previous studies have not analyzed all these previous determinants simultaneously. Second, we find that studies of inequality neglect how technology, institutions, and geography may be affecting economic development and inequality differently.

## Empirical analysis

### Econometric specification and estimation strategy

Empirical approaches to study differences in income levels have been proposed in economics using growth dynamics, which brings to a debate between neoclassical economics and the endogenous growth theory. Neoclassical economics assumes that economic growth can be explained by external forces related to the combination of the factors of production, mainly capital and labor (Solow 1956). On the other hand, endogenous growth theory proposes an alternative explanation of economic growth through endogenous drivers, such as human capital, knowledge, and innovation (Romer 1994). Both theories have emphasized the combination of physical capital, human capital, and technology, together with a residual that captures other factors that cannot be explained. Institutional quality may be helpful to explain this residual (Rodríguez-Pose and Ketterer 2020; Rodríguez-Pose 2020).

For this study, we use a generalized production function approach (e.g., Zellner and Revankar 1969), which allows us to measure the impact that technology, geography, and institutions exert on regional development. We present our baseline model in Eq. (1). This equation is theoretically and empirically rigorously founded since we follow the common generalized production functions together with the augmented Solow model from Mankiw et al. (1992). Both cases propose a specification where the logarithm of GDP per capita in levels is the dependent variable. Besides, we also follow the empirical specification from Rodríguez-Pose and Ketterer (2020) to include other geographical, institutional, and economic determinants. In contrast to previous studies, we extend the empirical model incorporating ICT variables.

1 $$\begin{aligned} \ln {\text{GDPpc}}_{it} = & \beta_{0} + \beta_{1} \ln {\text{HHINT}}_{it} + \beta_{2} \ln {\text{HHBR}}_{it} + \beta_{3} \ln {\text{EMPLICT}}_{it} + \beta_{4} {\text{QoG}}_{it} \\ & \quad + \beta_{5} {\text{HDD}}_{it} + \beta_{6} \ln {\text{ACCESS}}_{i} + \beta_{7} \ln {\text{TRI}}_{i} + \beta_{8} \ln {\text{INV}}_{i,t - 1} \\ & \quad + \beta_{9} \ln {\text{POP}}_{it} + \beta_{10} \ln {\text{EDUC}}_{it} + \varepsilon_{it} \\ \end{aligned}$$

where ln denotes the natural logarithm and subscripts *i* and *t* refer to region and time, respectively. ${\mathrm{GDPpc}}_{it}$ is the regional income per capita in region *i* at time *t*. ${\mathrm{HHINT}}_{it}$ denotes the percentage of households having access to the Internet, ${\mathrm{HHBR}}_{it}$ is the percentage of households with broadband access, ${\mathrm{EMPLICT}}_{it}$ is the share of employment in the Information and Communications Technology (ICT) sector, ${\mathrm{QoG}}_{it}$ is the regional quality of government, ${\mathrm{HDD}}_{it}$ is the number of heating degree days, ${\mathrm{ACCESS}}_{i}$ is the accessibility index, and ${\mathrm{TRI}}_{i}$ is the average terrain ruggedness index in the region. ${\mathrm{INV}}_{it-1}$ denotes the 1-year lag of investment, and ${\mathrm{POP}}_{it}$ is population in the region ${\mathrm{EDUC}}_{it}$ is human capital. Finally, ${\varepsilon }_{it}$ denotes the error term.

Growth and economic development theories have incorporated technology as an explanatory variable by assuming that technology increases efficiency and productivity (Basu and Weil 1998). Despite the relevance of ICT as a regional growth driver in the European Union, existing literature on the effects of ICT is scarce and has only focused on a single variable. For this reason, we use three variables to measure ICT. First, the share of the ICT sector in the regional gross value added to capture the size of the ICT sector in the economy, as in Martínez and Rodríguez (2009). Although the share of this sector in the economy depends on the region production structure itself, descriptive statistics in Table A.2 show that lagging regions have a small share. A traditionally agricultural region would have a smaller share of the ICT sector. However, it might have higher levels of economic development and less need for ICT convergence in the production share of the economy. Therefore, it is necessary to complement this variable with other indicators of ICT penetration in the region. Second, we follow other studies (Vicente and López 2011; Billón et al. 2017) by using ICT variables related to households: the percentage of households with access to the Internet—to measure the quantity—and the percentage of households with broadband access—as a measure of the quality (speed).

In the European Union, Internet access has become relatively cheap due to the high competition in the market of Internet service providers as a result of liberalizing market access. Therefore, one might think that the percentage of households with access to the Internet might not be a good proxy for digital technology in explaining growth, being speed a more relevant proxy. However, Fernandez-Portillo et al. (2020), using data for the OECD European countries, find that the number of Internet users is the ICT indicator with the highest performance on explaining GDP growth. We consider relevant to include both the share of households with access to the Internet and households with broadband access as a proxy for the speed.

Intrinsically related to technology, human capital has also been demonstrated to be important for increasing economic growth since differences in education can help to explain differences in income per capita (Mankiw et al. 1992) and this pattern is also observed at the regional level (Gennaioli et al. 2014).

Geography has also been recognized as a growth driver (Sachs et al. 2001), but finding a suitable variable to measure the impact of geography has been subject to debate by academic scholars. Albalate et al. (2021) find that temperature, measured through the heating degree days, is the most important factor explaining population density in European regions. Using a model that predicts the regional population distribution from geographical factors, they find that misadjustment between observed and predicted population distribution harms economic growth. A rugged terrain may make it difficult to develop certain living conditions (Nunn and Puga 2012) and be negatively associated with growth and development. Lower accessibility and higher ruggedness increase the cost of transport and trade (Ketterer and Rodríguez-Pose 2018). Regions with lower accessibility can find in ICT a solution to overcome the curse of geography. However, greater ruggedness increases the cost of deploying the necessary technological infrastructure.

Growth theory has demonstrated that a combination of the aforementioned factors is not sufficient to explain growth patterns. Although growth theory has been continuously improving, regional growth patterns show an increasing residual factor that suggests the existence of missing elements (Rodríguez-Pose and Ketterer 2020). Among all the potential elements that could trigger regional growth, the role of institutions is found to be a major regional growth driver, especially after the progress made in measuring the quality of government at the subnational level in the European Union (Charron et al. 2019). A positive effect is expected since those regions with better quality institutions register higher levels of regional growth (Ketterer and Rodríguez-Pose 2018; among others).

One of the most critical aspects of this article is how to measure inequality, given the importance of this variable to explain research objectives and the heterogeneity of potential indicators.^5^ We begin with the concept of social exclusion, coined in Sociology, as this is a broader concept that may comprise inequality. Lenoir (1974) defined social exclusion as a new source of inequality that prevents selected individuals or social groups from their full participation in society. As poverty has been considered as the main indicator to measure social exclusion by institutions and national bureaus of statistics, we hypothesize that risk of poverty may accurately capture differences in regional inequality accurately, in contrast to other variables like the Gini Index, which is the most widely used indicator to measure inequality but has been subject to criticism.^6^

The lack of social variables could also be mentioned as another factor that has increased the growth residual. To overcome this issue, following Perugini and Martino (2008), we define a second Eq. (2) to study whether the previous determinants that explain economic development can also explain the risk of poverty and social exclusion in European regions^7^:

2 $$\begin{aligned} \ln {\text{RISKP}}_{it} = & \beta_{0} + \beta_{1} \ln {\text{HHINT}}_{it} + \beta_{2} \ln {\text{HHBR}}_{it} + \beta_{3} \ln {\text{EMPLICT}}_{it} + \beta_{4} \ln {\text{QoG}}_{it} \\ & \quad + \beta_{5} {\text{HDD}}_{it} + \beta_{6} \ln {\text{ACCESS}}_{i} + \beta_{7} \ln {\text{TRI}} + \beta_{8} \ln {\text{INV}}_{i,t - 1} + \beta_{9} \ln {\text{POP}}_{it} \\ & \quad + \beta_{10} \ln {\text{EDUC}}_{it} + \varepsilon_{it} \\ \end{aligned}$$

where $\mathrm{ln}{RISKP}_{it}$ is the logarithm of the risk of poverty and social exclusion in a specific region. The remaining variables are as in Eq. (1).

Whether the results are driven by endogeneity due to the possible correlation of the percentage of households with access to Internet and broadband, the share of the ICT sector in the regional economy, and the institutional quality variables with the unobserved individual random effect is an important fact to address. To this end, the existence of potential problems of endogeneity between economic development and technology (Grossman and Helpman 1991), geography (Sachs et al. 2001), and institutions (Glaeser et al. 2004) has been identified in previous studies. As investment is included in the measure of GDP, we include the first lag of the investment variable to overcome the possible problem of endogeneity.

### Data

The analysis is based on a balanced panel of 229 NUTS-2 level regions for the 27 European Union countries, excluding the UK, during 2007–2018. Data on the gross domestic product, population, and investment—gross capital formation—come from the ARDECO database. Education, households’ access to the Internet and broadband access, the risk of poverty and social exclusion, and the share of employment in the ICT sector are taken from The Quality of Government EU Regional Dataset (Charron et al. 2020) and the Eurostat Regional Statistics database. The exact definitions of the variables and data sources used are summarized in Table A.1 in the Appendix. Descriptive statistics are presented in Table A.2. Quality of government data is taken from Charron et al. (2021), which used survey data on citizens’ perceptions and experiences of public sector corruption, impartiality, and public sector services quality.

The terrain ruggedness index (TRI) is computed following Riley et al. (1999) and Wilson et al. (2007) using the European Digital Elevation Model (EU-DEM) provided by the Copernicus Programme of the European Environmental Agency. The average TRI for each region is computed by overlapping the computed TRI raster layer with the polygon vector layer of the NUTS-2 regions provided by Eurostat GISCO.

## Results

Estimation results of the regional economic development Eq. (1) are presented in Table 2 using different estimation methods. Columns (I) and (II) present estimation results for the random effects model. The Breusch and Pagan (1980)—Lagrange multiplier test for random effects rejects the null hypothesis that the variance of the random effects is zero, implying that random effects are significant. As some of our explanatory variables are time invariant—Accessibility and Ruggedness, the Hausman test to check for fixed effects or random effects is not appropriate because these variables would be dropped from the fixed effects regression. The Wooldridge test of serial correlation reveals the existence of first-order autocorrelation. To correct for the presence of serial correlation, we present in columns (III) and (IV) the estimation results of generalized random effects (GLS) with correlated panels and a first-order autoregressive disturbance AR (1) model.

**Table 2:** Economic development equation estimation results

	(I)	(II)	(III)	(IV)	(V)	(V)
	RE	RE	GLS	GLS	HT	HT
$\mathrm{ln}Internet$	0.169***		0.229***		0.149***	
	(0.020)		(0.071)		(0.021)	
$\mathrm{ln}Broadband$		0.080***		0.011		0.072***
		(0.013)		(0.040)		(0.014)
$\mathrm{ln}Share ICT$	0.059***	0.064***	0.054	0.082*	0.006	0.007
	(0.014)	(0.015)	(0.048)	(0.047)	(0.015)	(0.015)
$Institutions$	0.121***	0.125***	0.307***	0.314***	0.079***	0.080***
	(0.012)	(0.012)	(0.037)	(0.044)	(0.013)	(0.013)
$\mathrm{ln}HDD$	− 0.102***	− 0.102***	− 0.050*	− 0.074***	− 0.090**	− 0.089**
	(0.036)	(0.037)	(0.026)	(0.026)	(0.037)	(0.038)
$\mathrm{ln}Accessibility$	0.277***	0.300***	0.008	0.075	0.370***	0.396***
	(0.047)	(0.048)	(0.060)	(0.075)	(0.060)	(0.062)
$\mathrm{ln}Ruggedness$	0.085***	0.089***	0.041	0.060***	0.065***	0.066***
	(0.018)	(0.019)	(0.053)	(0.017)	(0.023)	(0.024)
$Lag \mathrm{ln}Investment$	0.352***	0.344***	0.303***	0.293***	0.284***	0.274***
	(0.018)	(0.018)	(0.039)	(0.039)	(0.019)	(0.018)
$\mathrm{ln}Population$	− 0.357***	− 0.349***	− 0.281***	− 0.280***	− 0.358***	− 0.372***
	(0.024)	(0.024)	(0.054)	(0.047)	(0.035)	(0.039)
$\mathrm{ln}Human Capital$	0.206***	0.254***	0.025	0.209**	0.224***	0.268***
	(0.026)	(0.027)	(0.060)	(0.097)	(0.028)	(0.028)
$Constant$	11.568***	11.784***	10.608***	11.126***	12.276***	12.761***
	(0.458)	(0.465)	(0.634)	(0.568)	(0.659)	(0.712)
Observations	2,748	2,748	2,748	2,748	2,748	2,748
Number of regions	229	229	229	229	229	229
Breusch–Pagan test	5272.89***	5071.43***				
Wooldridge test	210.33***	222.58***				
Underidentification					141.035***	139.637***
Weak identification					1401.645***	1567.232***
R-squared	0.888	0.877	0.831	0.831	0.818	0.787
Wald Chi2	1619.15***	1465.72***	1141.44***	659.07***	268,840.22***	257,314.30***

Cluster-robust standard errors in parenthesis

*RE* random effects, *GLS* generalized random effects with correlated panels and a first-order autoregressive disturbance AR(1), *HT* Hausman and Taylor. R-squared in the GLS and HT estimations is computed as the squared correlation coefficient between observed and predicted values. The dependent variable is the log of regional GDP per capita. Underidentification test corresponds to the Kleibergen–Paap LM statistic, while weak identification corresponds to the Kleibergen–Paap Wald F statistic

***, and **, and * denote significance at the 0.01, 0.05, and 0.10 levels, respectively

In addition, in columns (V) and (VI), we present the results of a Hausman and Taylor (HT) estimation. The Hausman and Taylor (1981) model is an intermediate position between fixed effects and random effects based on instrumental variables. Endogenous time-varying variables are instrumented using a within transformation of the exogenous time-varying variables, while endogenous time-invariant regressors are instrumented from the individual means of the exogenous time-varying variables. Such an instrumentation procedure relieves from resorting to external variables to be used as instruments, which is appropriate in our context as data availability at the subnational level is relatively scarce compared to the country level. The random effects GLS with AR(1) and the HT estimators have already been used in tandem in the literature at the regional level and results are consistent (e.g., Albalate et al. 2012).

The HT estimator allows us to include the time-invariant accessibility and ruggedness variable in our model while controlling for possible endogeneity of the access to the Internet and broadband variables, the share of employment in the ICT sector, and the institutional quality. Consistent estimation of the HT model requires that the number of exogenous time-varying variables be equal to or larger than the number of endogenous time-invariant variables. As all our time-invariant variables are geographical, none is considered endogenous and this condition is met. The Kleibergen and Paap (2006) rank LM statistic for underidentification shows that the model is identified, while the Kleibergen–Paap Wald F statistic robust to heteroskedasticity and clustering rejects the null hypothesis of weak identification.

The estimated coefficients for the percentage of household with access to the Internet are positive and significant in all models, while the percentage of households with broadband access is significant in the RE and HT models but not in the GLS. The estimated coefficients for the broadband variable are around two times lower than for the access to the Internet variable. These results confirm the importance of distinguishing between the quantity and quality of ICT, as indicated by Hilbert (2016), since their contribution to regional growth differs significantly. The share of employment in the ICT sector is positive but not significant when controlling for serial correlation or endogeneity. The quality of institutions is positive and statistically significant, in line with previous studies that find that good quality of government is associated with regional growth (e.g., Rodríguez-Pose and Ketterer 2020). Investment, human capital, and population estimated coefficients align with the literature estimating an extended version of Mankiw et al. (1992), with positive effects for investment and human capital and negative for population.

When examining the geographical variables, we find that the estimated coefficients for heating degree days are negative and significant, indicating that cold weather may be harmful to economic development, as more resources are needed to heat the buildings and keep the population within a healthy temperature. This is particularly relevant in the current context, where average temperatures show an uprising trend over time.

Accessibility is positive and significant, indicating that central regions benefit from being closers to the market while having lower transportation costs. Despite increasing trade costs and the cost of building transport infrastructure, ruggedness has a positive and significant effect on income.

The existence of nonsignificant coefficients in the GLS AR(1) model can be explained because the effect of certain variables may be captured by others, as explained by Ketterer and Rodríguez-Pose (2018), when studying the complementarity between geography and institutions to explain economic growth. In our case, digital technologies and institutional quality are overriding the importance of geographical factors. Moreover, the presence of peripheral regions in the analysis may undermine certain growth drivers since their factor endowment tends to be lower. Finally, we find that coefficients are fairly stable across estimations.

Estimation results for the risk of poverty Eq. (2) are presented in Table 3. The results show how regional social exclusion is negatively associated with ICT, with the share of households with access to the Internet and the share of employment in the ICT sectors being more important than broadband access. Although ICT variables are nonsignificant for the GLS AR(1) estimation, they are significant in the HT estimation that controls for endogeneity. The coefficients are also negative for other regional variables, such as investment, human capital, and institutional quality. To sum up, social exclusion only maintains a positive relationship with population. Most of the factors that are positively associated with economic growth are also related to a lower risk of social exclusion. Geography reveals to be less important for explaining the risk of poverty and social exclusion, with heating degree days and ruggedness being not significant when controlling for serial correlation or endogeneity. Accessibility is negative and significant in all models, indicating that being distant from other markets and the physical EU core is associated with a higher risk of poverty and social exclusion.

**Table 3:** Risk of poverty and social exclusion equation estimation results

	(I)	(II)	(III)	(IV)	(V)	(V)
	RE	RE	GLS	GLS	HT	HT
$\mathrm{ln}Internet$	− 0.086***		− 0.070		− 0.065***	
	(0.020)		(0.084)		(0.020)	
$\mathrm{ln}Broadband$		− 0.021*		− 0.037		− 0.006
		(0.012)		(0.045)		(0.012)
$\mathrm{ln}Share ICT$	− 0.041**	− 0.046***	− 0.040	0.052	− 0.035*	− 0.041**
	(0.017)	(0.018)	(0.058)	(0.053)	(0.019)	(0.020)
$Institutions$	− 0.096***	− 0.097***	− 0.101*	− 0.081*	− 0.097***	− 0.097***
	(0.017)	(0.017)	(0.058)	(0.047)	(0.019)	(0.020)
$\mathrm{ln}HDD$	− 0.034***	− 0.033**	0.030	− 0.022	− 0.022	− 0.022
	(0.013)	(0.013)	(0.067)	(0.061)	(0.016)	(0.016)
$\mathrm{ln}Accessibility$	− 0.199***	− 0.219***	− 0.792***	− 0.665***	− 0.237***	− 0.262***
	(0.046)	(0.046)	(0.274)	(0.175)	(0.071)	(0.080)
$\mathrm{ln}Ruggedness$	− 0.061***	− 0.065***	− 0.118	0.064	− 0.042	− 0.042
	(0.016)	(0.016)	(0.134)	(0.082)	(0.027)	(0.031)
$Lag \mathrm{ln}Investment$	− 0.209***	− 0.198***	− 0.157***	− 0.201***	− 0.212***	− 0.202***
	(0.016)	(0.016)	(0.057)	(0.056)	(0.017)	(0.017)
$\mathrm{ln}Population$	0.253***	0.245***	0.067	0.422**	0.551***	0.612***
	(0.027)	(0.027)	(0.153)	(0.176)	(0.070)	(0.080)
$\mathrm{ln}Human Capital$	− 0.022	− 0.078**	− 0.096	− 0.184*	− 0.071*	− 0.134***
	(0.036)	(0.034)	(0.106)	(0.109)	(0.039)	(0.038)
$Constant$	1.935***	1.839***	3.033	− 1.113	− 2.335**	− 3.360***
	(0.318)	(0.328)	(2.219)	(2.624)	(0.938)	(1.077)
Observations	2,748	2,748	2,748	2,748	2,748	2,748
Number of regions	229	229	229	229	229	229
Breusch–Pagan test	9832.81***	9608.67***				
Wooldridge test	75.487***	74.683***				
Underidentification					141.095***	139.670***
Weak identification					2540.403***	2903.127***
R-squared	0.550	0.538	0.428	0.377	0.330	0.279
Wald Chi2	466.45***	417.13***	37.24***	44.81***	23,714.28***	18,452.23***

Cluster-robust standard errors in parenthesis

*RE* random effects, *GLS* generalized random effects with correlated panels and a first-order autoregressive disturbance AR(1), *HT* Hausman and Taylor. R-squared in the GLS and HT estimations is computed as the squared correlation coefficient between observed and predicted values. The dependent variable is the log of the risk of poverty and social exclusion. Underidentification test corresponds to the Kleibergen–Paap LM statistic, while weak identification corresponds to the Kleibergen–Paap Wald F statistic

***, and **, and * denote significance at the 0.01, 0.05 and 0.10 levels, respectively

These results point to the importance of considering inequality parallel to growth factors when examining regional growth patterns and designing regional policy. Factors that explain the observed rising income inequality in recent years are characteristics of peripheral regions, such as greater levels of deprivation of economic activity and higher inequalities (Rodríguez-Pose 2018). Investing in ICT technologies and human capital, promoting the technological sector, and improving the quality of institutions are recipes to reduce the risk of poverty and social exclusion. These factors will undoubtedly determine the aggregate outcomes and future prospects of the regions.

## Conclusions

In this study, we have analyzed the importance of technological, institutional, and geographical determinants simultaneously to explain income determinants for a sample of 229 European regions during the period 2007–2018. We also extend this analysis to inequality to check whether the previous explanatory factors can be considered as drivers of the risk of poverty and social exclusion. The results show that information and communication technologies are positive and significant to explain regional development, but it is important to distinguish between quantity and quality. Institutional quality and some geographical variables are also positive and significant. In particular, we find that population access to the Internet and a greater weight of the ICT sector in the economy are tools for dealing with rising levels of inequality, as regional social exclusion is inversely related to the size of the technological sector, good government institutions, and investment.

Our results suggest that the interplay between technology, institutional quality, and geography may be important to explain regional development and risk of poverty in the European Union and demands to be considered for the curse of action when planning regional policies. Place-based policies should consider geographical characteristics of the territories, such as worse accessibility and higher ruggedness, because they increase the cost of building infrastructure and lead to the isolation of the population.

By considering the importance of place-based policies, the Cohesion Policy has led to remarkable initiatives to reduce regional inequalities. Investing in transport infrastructure to connect peripheral regions to the core Europe has been the recipe for many decades, and the Cohesion Policy has dedicated an enormous amount of money to this endeavor. Building new roads, highways, and railways have been the priority of many countries to improve their accessibility, and the Trans-European Transport Network (TEN-T) project aims to build a Europe-wide network connecting all the main European nodes by 2030, and all European regions by 2050. Investments in transport infrastructure help in reducing the peripheral nature of the most distant regions. However, in the context of a New Globalization where the ICT revolution is completely reshaping the economy through disruptive changes (Baldwin 2016), a new framework of regional policy is desirable. A technological infrastructure policy is needed to connect all European regions through high-speed broadband connections.

Social exclusion is also an important problem to overcome. Although the Europe 2020 strategy aims to reduce the risk of poverty as its main priority, greater amounts of social investment may be required to reduce regional disparities. However, these investments need to be complemented with high-quality institutions that serve as catalysts and boost regional economic development and prosperity. We confirm that the interplay between technology, institutions, and geography is expected to reshape economic performance in the following decades. They cannot be considered as substitutes but complements.

An effective regional planning policy may be desirable to overcome the aftermath derived from COVID-19 and mitigate this shock. In the European Union, this is particularly prominent after COVID-19, which undoubtedly requires finding drivers that enhance regional development while reducing social disparities. The Recovery and Resilience Facility implemented by the European Commission to help the European Union to speed up the recovery after the pandemic indicates that member states must expend at least 20% of the funds to foster the digital transition.

The main policy lesson that emerges from the empirical analysis indicates that greater investment in ICT and policies aiming to promote the digital literacy of the population could help in reducing social exclusion and foster economic development in declining or persistently stagnating regions. To this end, physical and social peripheries could take advantage of ICT to increase their digital connectivity with the rest of the world, attract new entrepreneurs, and bring prosperity to the regions. The curse of geography of being located far from the big economic players must not condemn regions to be disconnected from the digital world and remaining excluded in impoverished areas. For this reason, institutions can be considered as a key asset to trigger both public and private investments to enhance economic growth.

The contribution in this manuscript also suggests certain avenues for future research. The most prominent ones can be related to information technology and institutions. First, it would be convenient to incorporate emerging types of ICT into the analysis, given their growing importance in economic growth (Vu et al. 2020). Second, concerning the quality of institutions, it is necessary to extend the discussion not only to formal institutions (e.g., quality of government), but also to informal institutions related to cultural and familial ties, given the increasing interest shown by academic scholars in this topic (e.g., Tabellini 2010; Alesina and Giuliano 2015; Qayyum et al. 2020; Michalopoulos and Xue 2021). Thirdly, it would be desirable to examine the impact of digital technology on well-being, as Peiró-Palomino et al. (2020) do for the case of institutional quality, given the uprising interest in studying drivers of social welfare. Finally, extending the empirical model to consider possible spatial interaction effects between regions is interesting for future research.

## Supplementary Information

Below is the link to the electronic supplementary material.

Supplementary file1 (PDF 690 KB)

## Footnotes

1 See Appendix 2 for additional figures depicting the relationship between household access to broadband, GDP per capita, and risk of poverty or social exclusion.

2 We follow the classification of institutions proposed by Acemoglu and Robinson (2012).

3 See Table A1 in the Appendix for a description of the variables and the data sources.

4 Since the number of studies at country level is relatively abundant, we prefer to grasp existing literature at regional level.

5 Regional statistics are scarce in the European Union, and the European Social Survey (ESS) has been used as the main source of social regional statistics. However, the ESS indicators are not intended to capture regional perceptions, but country-level perceptions.

6 See, for instance, Gastwirth (2017).

7 Instead of the risk of poverty as the dependent variable, Perugini and Martino (2008) use the Gini index and the 90/10 percentile ratio of household income.
