# Supplementary material for: Technological, institutional, and geographical peripheries: regional development and risk of poverty in the European regions
Source: Ann Reg Sci. 2022 Apr 2;69(2):311–32. doi: 10.1007/s00168-022-01127-9 (PMC8976460; doi:10.1007/s00168-022-01127-9)

Supplemental Online Appendix

Technological, institutional, and geographical peripheries: regional development and risk of poverty in the European regions

Appendix 1: Data

Table A.1: Description of Variables.

**Variable**	**Definition**	**Data source**
Gross domestic product per capita	Gross domestic product (GDP) at current market prices per capita	ARDECO
Risk of Poverty and Social Exclusion	Percentage of people at risk of poverty or social exclusion	Eurostat
Households Access to the Internet	Percentage of households with access to the Internet at home	Eurostat
Households Broadband	Percentage of households with broadband access	Eurostat
Share of employment in the ICT sector	Percentage of employment in the Information and Communication sector (NACE-2 sector J)	Eurostat
Institutions	European Quality of Government Index (EQI)	Charron et al. (2021)
Heating Degree Days	Technical index describing the energy requirements of buildings in terms of heating	Eurostat
Accessibility	Sum of the inverse of distances to all NUTS-2 regions	Eurostat GISCO
Ruggedness	Terrain Ruggedness Index (Riley et al., 1999; Wilson et al., 2007)	EU-DEM 1.0
Investment	Gross fixed capital formation	ARDECO
Population	Population on 1 January	ARDECO
Human Capital	Percentage of population aged 25-64 with tertiary education (ISCED-2011 levels 5 to 8)	Eurostat

As the Quality of Government index is available only for 2010, 2013, 2017, and 2021, the middle years are interpolated. For the years before 2010, we assume that regional quality of governments difference with respect to the national quality of government is kept constant. National quality of government indexes are constructed as an unweighted average of the Control of Corruption, Government Effectiveness, Rule of Law, and Voice and Accountability indicators of the

Worldwide Governance Indicators, as it is standard in the literature (Rodríguez-Pose and Ketterer, 2019).

Eurostat data is taken from The Quality of Government EU Regional Dataset (Charron et al., 2020).

Due to data limitations, we exclude from the analysis:

- The Spanish autonomous cities of Ceuta (ES63) and Melilla (ES64).
- The Finish region of Pohjois- ja Itä-Suomi (FI1D).
- The French overseas territories: Guadeloupe (FRY1), Martinique (FRY2), Guyane (FRY3), La Réunion (FRY4), and Mayotte (FRY5).
- The Portuguese autonomous regions: Região Autónoma dos Açores (PT20) and Região Autónoma da Madeira (PT30).
- The Polish region of Mazowiecki regionalny (PL92).

For some countries, data for some variables are not available for all NUTS-2 regions for some years. We follow a data imputation procedure in those cases to build a balanced panel. First, we interpolate the data for the missing years, and we extrapolate it assuming that all NUTS-2 regions evolve with the same patterns as their corresponding NUTS-1 region, and NUTS-1 regions with their corresponding country. We have checked that the mean and standard error of the variables are similar before and after the imputation procedure.

Table A.2: Descriptive Statistics.

	Mean	Std. Dev.	Min	Max.
GDP per capita (€)	25,809.381	13,492.716	2,905.093	99,755.117
Risk of Poverty (%)	24.342	9.480	7.100	73.843
Households Internet (%)	71.728	16.631	12.920	100.000
Household Broadband (%)	65.913	18.758	5.353	100.000
Share Employment ICT (%)	2.440	1.525	0.333	9.200
Institutions	0.116	0.990	-2.796	2.818
Heating Degree Days	2,684.682	966.346	19.760	7,184.913
Accessibility	0.378	0.131	0.094	0.694
Ruggedness	2.146	1.858	0.130	10.324
Investment (Million €)	10,641.134	13,402.393	234.260	169,294.594
Population (Number)	1,901,292.779	1,596,531.803	26,923.000	12,219,796.000
Human Capital (%)	26.253	8.959	6.800	58.400

Note: 2,748 observations for 229 regions and 12 years.

Appendix 2: Additional figures

Figure A1. Percentage of households with broadband access to the Internet in 2018

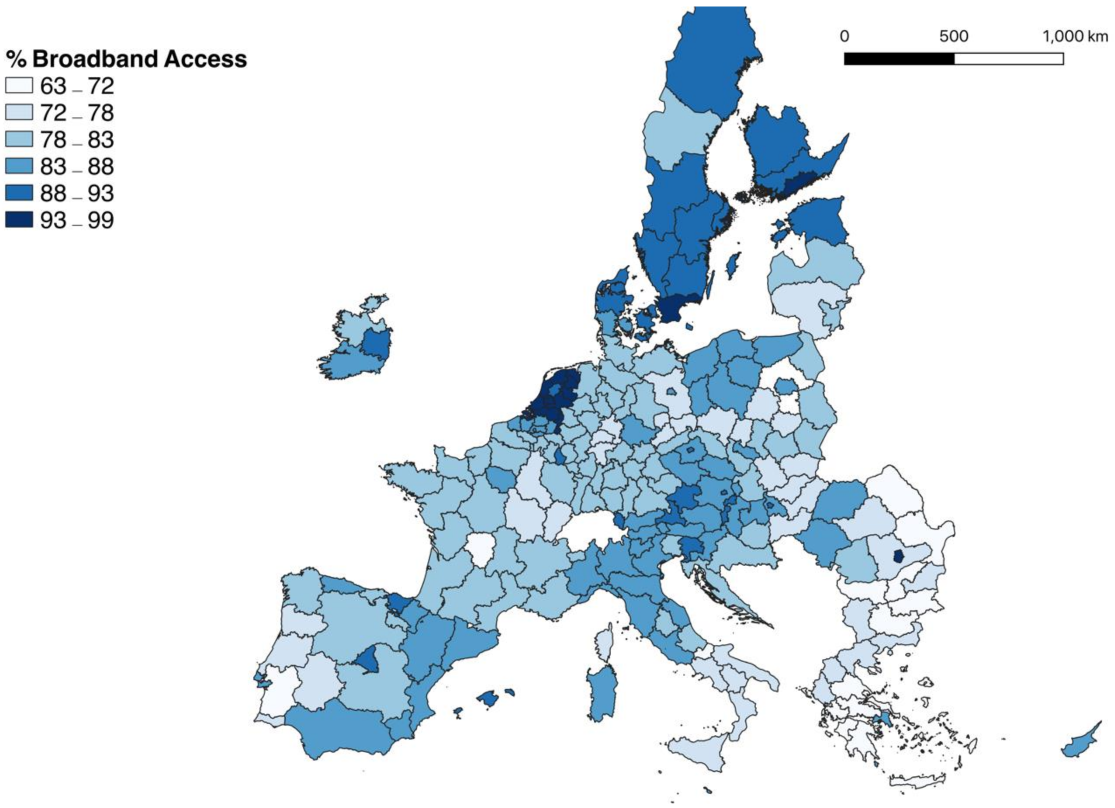

Source: Eurostat. EuroGeographics for the administrative boundaries. Note: The continental EU regions of Pohjois- ja Itä-Suomi (Finland) and Mazowiecki regionalny (Poland) are excluded.

Figure A2. Log of Percentage of households with broadband access to the Internet and log of GDP per capita in 2018

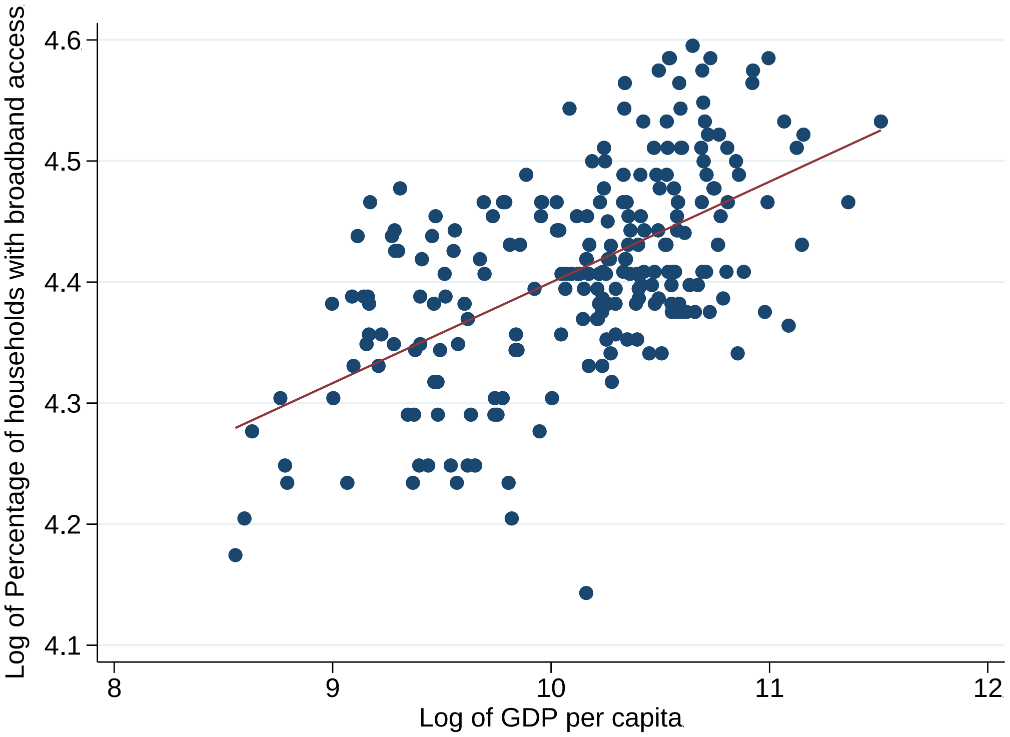

Figure A3. Percentage of households with broadband access to the Internet and Percentage of people at risk of poverty or social exclusion in 2018

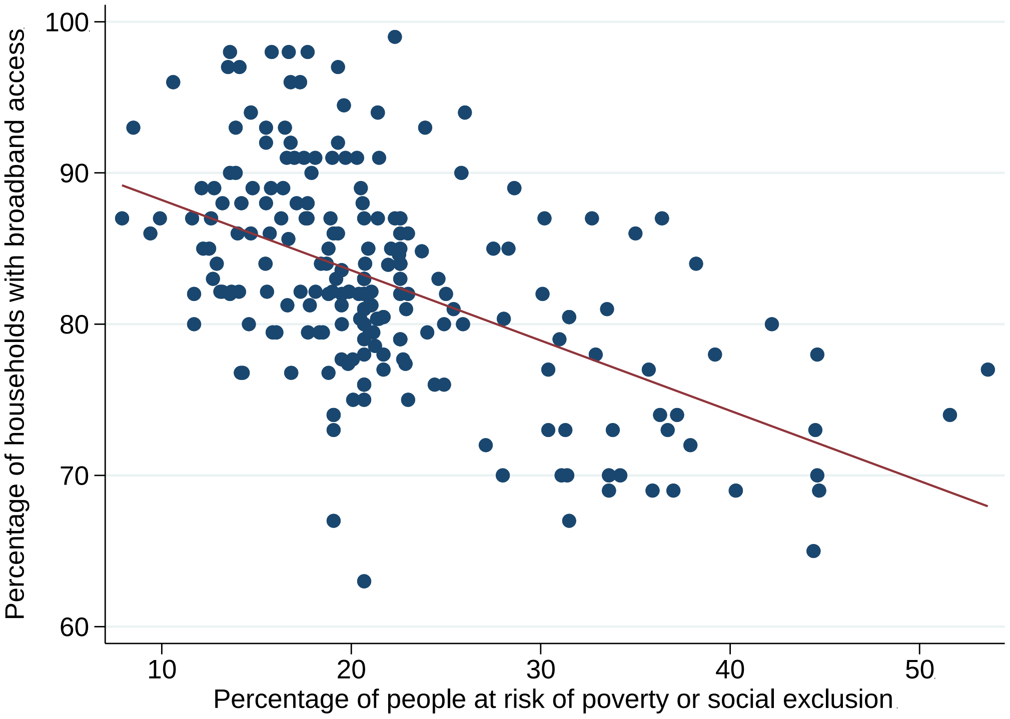

Supplement: Supplementary file 1 — Supplementary file1 (PDF 690 KB) [file 168_2022_1127_MOESM1_ESM.pdf]
